# Water column gradients beneath the summer ice of a High Arctic freshwater lake as indicators of sensitivity to climate change

**DOI:** 10.1038/s41598-021-82234-z

**Published:** 2021-02-03

**Authors:** Paschale N. Bégin, Yukiko Tanabe, Milla Rautio, Maxime Wauthy, Isabelle Laurion, Masaki Uchida, Alexander I. Culley, Warwick F. Vincent

**Affiliations:** 1 Centre d’études nordiques, Quebec City, QC Canada; 2 Département de biologie and Takuvik Joint International Laboratory, Université Laval, Quebec City, QC Canada; 3 National Institute of Polar Research, Tachikawa, Japan; 4 Département des sciences fondamentales, Université du Québec à Chicoutimi, Chicoutimi, QC Canada; 5 Centre Eau Terre Environnement, Institut national de la recherche scientifique, Quebec City, QC Canada; 6 Département de biochimie, de microbiologie et de bio-informatique and Takuvik Joint International Laboratory, Université Laval, Quebec City, Canada

**Keywords:** Freshwater ecology, Climate sciences, Ecology, Environmental sciences, Limnology

## Abstract

Ice cover persists throughout summer over many lakes at extreme polar latitudes but is likely to become increasingly rare with ongoing climate change. Here we addressed the question of how summer ice-cover affects the underlying water column of Ward Hunt Lake, a freshwater lake in the Canadian High Arctic, with attention to its vertical gradients in limnological properties that would be disrupted by ice loss. Profiling in the deepest part of the lake under thick mid-summer ice revealed a high degree of vertical structure, with gradients in temperature, conductivity and dissolved gases. Dissolved oxygen, nitrous oxide, carbon dioxide and methane rose with depth to concentrations well above air-equilibrium, with oxygen values at > 150% saturation in a mid-water column layer of potential convective mixing. Fatty acid signatures of the seston also varied with depth. Benthic microbial mats were the dominant phototrophs, growing under a dim green light regime controlled by the ice cover, water itself and weakly colored dissolved organic matter that was mostly autochthonous in origin. In this and other polar lakes, future loss of mid-summer ice will completely change many water column properties and benthic light conditions, resulting in a markedly different ecosystem regime.

## Introduction

Winter ice cover is a key feature of north temperate and high latitude lakes and has a controlling effect on underwater light and gas exchange with the atmosphere. It can prevent complete mixing of the water column, thereby allowing stable physicochemical gradients to develop that in turn shape the biology of the lake ecosystem^1^. In high latitude lakes, the ice-cover season may extend over most of the year, and in Antarctica and colder parts of the High Arctic perennial or multi-year ice may persist throughout summer^2^. Major changes in extent, thickness and duration of summer ice are now being observed in the High Arctic associated with climate warming^3,4^, and are also predicted for certain lakes in Antarctica that are currently covered by thick perennial ice^5^. There is a pressing need to define and understand the current state of water column properties of perennially ice-covered lakes before these ecosystems shift to summer ice-free conditions.

**Figure 1:**
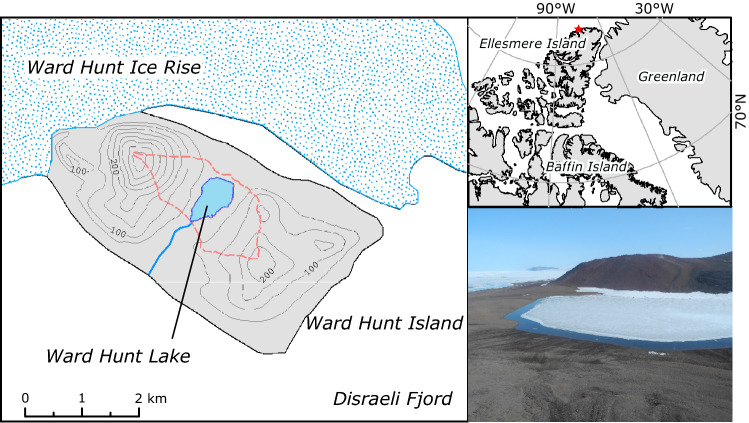
Ward Hunt Island, Canada. The map of the island (red star in location map) shows the lake and its watershed (red dashed line). The photograph shows the mid-summer (July) ice cover and the barren polar desert catchment. Shapefiles obtained via https://atlas.gc.ca/.

Ward Hunt Lake, Canada’s northernmost lake (Fig. 1), was covered by thick perennial ice for at least five decades, with the first measurement of 4.24 m thickness in mid-summer (July) 1954. Thinning of its ice-cover was reported in 2009, and complete disappearance of its ice for the first time in fall 2011^3^. This lake is entirely freshwater and thereby differs from other waterbodies along the adjacent northern coastline of Ellesmere Island, notably lakes A, B, C1, C2 and C3. These coastal lakes are deep and meromictic, characterized by saline bottom waters that do not mix with the surface freshwater layer^6^. Similarly, many of the well-known lakes of continental Antarctica, such as those in the McMurdo Dry Valleys, are meromictic with strong salinity gradients^7^. Less is known about perennially ice-covered polar freshwater lakes, and about their water column structure that could be perturbed by the loss of ice-cover.

The objectives of this study were to characterize the vertical structure of Ward Hunt Lake during the ice-covered summer period and to identify features that may be disrupted by ice loss and water column mixing. Given the vertical patterns that have been observed in seasonally ice-covered lakes with stable water layers located above and below solar driven convection cells^8^, our central hypothesis was that ice-cover presence and persistence would result in pronounced gradients in chemical, physical and biological properties, despite the lack of extreme salinity-density effects as observed in nearby meromictic lakes. We profiled Ward Hunt Lake at its deepest point, and examined changes in water chemistry with depth, including dissolved gases, the chlorophyll and carotenoid pigment stocks in the phytoplankton and benthic communities, and the fatty acid profiles of the planktonic communities. Finally, we applied a number of optical analyses to measure and interpret the changes in spectral irradiance down the water column. Given that the inputs of allochthonous carbon to the lake from the poorly developed soils in its barren polar desert catchment (Fig. 1; Supplementary Fig. S1) are likely to be small, we hypothesized that the underwater light regime would be controlled largely by the ice-cover, as well as by phytoplankton and water itself, with little contribution by colored dissolved organic matter (CDOM). To test this hypothesis, we characterized the DOM by spectral absorption and parallel factor fluorescence analysis (PARAFAC), and measured a set of apparent and inherent optical properties of the water column beneath the summer ice.

## Results

### Physicochemical profiles

**Figure 2:**
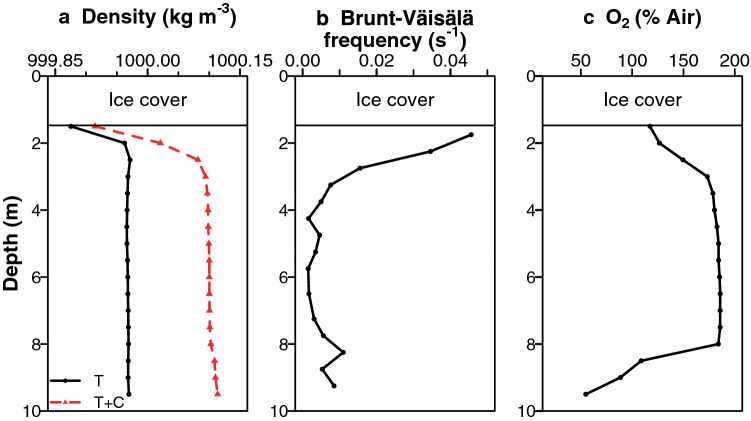
Physicochemical profiles in Ward Hunt Lake. (**a**) Density calculated considering temperature only (T; in black) and considering both temperature and salinity, as measured by conductivity (T + C; in red), (**b**) Brunt-Väisälä frequency and (**c**) dissolved oxygen (expressed as % of air equilibrium), 14 July 2016.

Ward Hunt Lake was covered by 2.18, 1.98 and 1.47 m of ice without snow cover when we sampled on 14 July of 2015, 2016 and 2017 respectively. Each year, an ice-free water zone (moat) forms on the northern and western shore of the lake and remains 10–20 m wide for most of the summer (Supplementary Fig. S1). The water column showed a pronounced stratification mainly driven by its dilute salinity gradients (Fig. 2). The buoyancy profile of Brunt-Väisälä frequency showed two stable water layers: a surface boundary layer just below the ice cover and a bottom boundary layer over the lake sediments. These density-stratified layers delimited an unstable stratum between 4 and 8 m (Fig. 2b) that contained homogeneous concentrations of dissolved oxygen at values well above saturation (Fig. 2c), suggesting a convection cell with high primary productivity. Inverse thermal stratification (warm water lying beneath cooler water) was observed in all mid-summer sampling profiles (Supplementary Fig. S2 for 2016 and 2017, with additional years shown in Supplementary Fig. S3a). Temperatures measured in the Ward Hunt Lake water column were higher in 2016, reaching 6.5 °C (Supplementary Fig. S2). The density gradients closely followed the specific conductivity profile (Fig. 2b,c), consistent with salinity control of stratification despite the low solute concentrations (dominated by Ca^2+^ and HCO_3_^−^; Supplementary Table S1). Dissolved O_2_ concentrations rose from near-equilibrium values immediately under the ice to well above saturation in the depth region 3 to 8 m, and declined to around 50% near the bottom of the lake (Supplementary Fig. S2d). This bottom layer also contained the highest concentration of Chl *a* (Supplementary Fig. S2e). For the more detailed profile in 2017 (Supplementary Fig. S2e), oxygen (O_2_) and Chl *a* concentrations were negatively correlated (Pearson’s correlation test, *r* = -0.71, df = 20, *p* < 0.001). Throughout most of the water column, Chl *a* values were in the range 0.4–0.7 µg L^−1^ (Supplementary Fig. S2e), indicative of oligotrophic conditions.

### Dissolved gases

**Figure 3:**
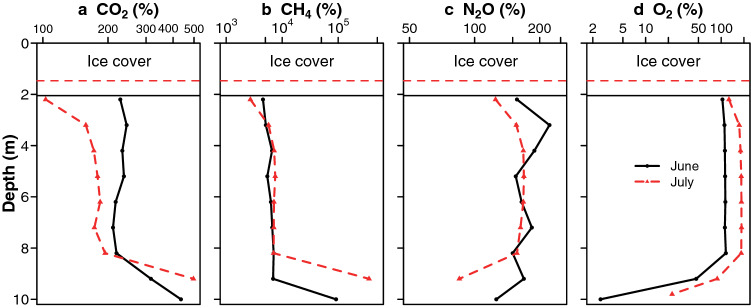
Concentrations of dissolved gases in Ward Hunt Lake on 7 June and 16 July 2017. (**a**) carbon dioxide (CO_2_), (**b**) methane (CH_4_), (**c**) nitrous oxide (N_2_O) and (**d**) oxygen (O_2_) expressed as % equilibrium with air, in Ward Hunt Lake in June (black) and July (red) 2017. Note the differences in logarithmic scales.

Concentrations of dissolved gases were well above atmospheric equilibrium below the ice cover, with maximum saturation values of 186% O_2_, 222% nitrous oxide (N_2_O), 497% carbon dioxide (CO_2_) and 355 thousand % methane (CH_4_). The concentrations of all four gases were homogenous in the mid-water column and then changed at lower depths, with divergent patterns: CO_2_ and CH_4_ increased sharply towards the bottom (Fig. 3a,b), whereas concentrations of N_2_O and O_2_ decreased (Fig. 3c,d). The CO_2_ and CH_4_ stored in the water column (from 2 to 9 m) dropped by half from June 7 to July 16, while N_2_O concentrations decreased by 19%; in contrast, dissolved O_2_ concentrations increased during this period, by 32% (Supplementary Table S2).

### Pigment stocks

Benthic mats coat the bottom of Ward Hunt Lake, and pigment analysis of benthic core samples taken in the present study confirmed the high biomass of these biofilm communities. This photosynthetic mat community was compared with the phytoplankton community by integrating the pigment concentrations for the water column and comparing pigments (Chl *a* and total carotenoids) per unit area. This showed that benthic microbial mats had pigment stocks that were were one to two orders of magnitude higher than the phytoplankton pigment stocks in the overlying water column (Table 1). There were large differences among the triplicate samples despite their proximity within the mid-lake area (radius of 15 m) and the same sampling depths.

**Table 1:** Pigments in the benthic mats and phytoplankton of Ward Hunt Lake.

	WH1	WH2	WH3
**Concentration (mg m**^**-2**^**)**			
Chl *a* benthic mats	841	65	148
Chl *a* water column^a^	3.7	2.7	3.4
Carotenoids benthic mats	642	29	99
Carotenoids water column^a^	2.6	2.3	2.7

Benthic core samples were from three mid-lake locations (WH1, WH2 and WH3) collected in July 2015. Total benthic pigment stocks per unit area are given for comparison with phytoplankton values integrated for the overlying water column at each site.

^a^Integration using the mean concentrations from three samples at each of the two depths: 2.2 and 7.5 m.

### Fatty acids

The fatty acid composition of water column seston was analyzed by principal coordinate analysis (PCoA) and showed a separation into upper and lower water column clusters (Supplementary Fig. S4; the main axis accounted for 54% of the variance). This separation was largely driven by differences in the fatty acid C16:1n-7, known to occur in cyanobacteria and diatoms, and C20:5n-3 (eicosapentanoenoic acid; EPA) and C22:6n-3 (docosadienoic acid; DHA), which are fatty acids common in many phytoplankton groups, including chrysophytes and dinoflagellates^9,10^. Linoleic and linolelaidic acids (regrouped in C18:2n-6) and alpha-linolenic acid (C18:3n-3), generally found in green algae^9^, were also present in the seston. The fatty acid assemblages for the total water column samples of zooplankton clustered along the first axis with seston from the upper water layer (Supplementary Fig. S4).

### Irradiance profiles

**Figure 4:**
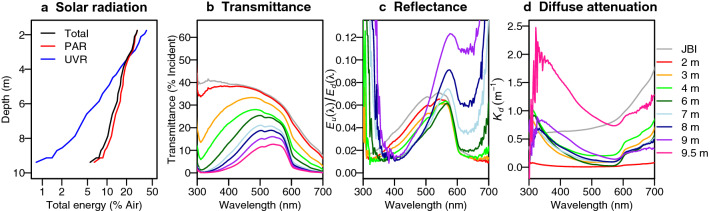
Underwater optical properties. (**a**) Downwelling irradiance for total shortwave, PAR and UVR; (**b**) transmittance as % downwelling incident irradiance in air; (**c**) reflectance as the ratio of upwelling to downwelling irradiance; and (**d**) diffuse attenuation coefficients under the summer ice of Ward Hunt Lake, 14 July 2016. The *K*_*d*_ values were plotted at *z*_*2*_; the *K*_*d*_ values for just below the ice (JBI) are ‘apparent attenuation coefficients’ because they were not corrected for reflection of incident light by the upper ice surface.

The ice-cover reflected and attenuated 71% of total incoming light energy, allowing 41% of ultraviolet radiation (UVR) and 29% of photosynthetically active radiation (PAR) to penetrate into the underlying water column (Fig. 4a). The total and PAR energy remained above 5% of incoming energy at the bottom of the lake, and UVR energy at the base of the water column was less than 1% incident UVR (Fig. 4a). At 3 m, the water column and ice had attenuated most light energy below 400 nm and above 600 nm and the spectrum shifted towards dominance by wavelengths around 550 nm (Fig. 4b), leaving mainly green light for the benthic microbial mat communities. Just below the ice, reflectance of light was higher within the range of 450–670 nm, whereas it shifted with a peak at 570 nm towards the bottom (Fig. 4c), consistent with the green-yellow hues seen in underwater videos from the lake^11^, and high values at wavelengths around 650 nm that may have been influenced by solar-induced chlorophyll *a* fluorescence in addition to the orange carotenoid-rich pigmentation of the benthic mats. The diffuse attenuation coefficient (*K*_*d*_) increased with depth until 4 m, decreased near 6 m and increased again to reach its highest values at 9.5 m, with an increase of attenuation at all wavelengths, but especially below 500 nm (Fig. 4d). The markedly higher *K*_*d*_ values at the bottom of the water column corresponded to the depth of highest concentrations of phytoplankton pigments (Supplementary Fig. S2).

### Optical properties of the lake water constituents

**Figure 5:**
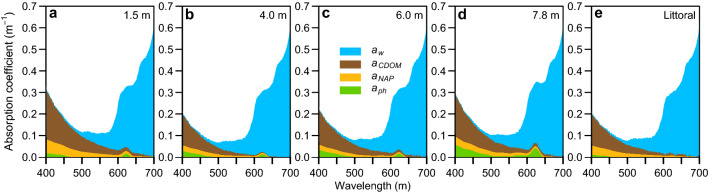
Spectral absorption by the optically active constituents in Ward Hunt Lake. The total absorption coefficients are partitioned according to: phytoplankton (*a*_*ph*_), non-algal particulates (*a*_*NAP*_), colored dissolved organic matter (*a*_*CDOM*_*)* and water molecules (*a*_*w*_). For comparison with four depths at the midlake station, data are also shown for surface waters of the littoral zone sampled on the same day.

Ward Hunt lake had low concentrations of dissolved and particulate matter, and light absorption in the offshore water column and in the littoral zone was mainly attributable to water itself (*a*_*w*_; Fig. 5a–e). At lower wavelengths, CDOM was the main light-absorbing component, with highest *a*_*CDOM*_ values just below the ice (1.5 m; Fig. 5a). When multiplied by the downwelling spectral irradiance at the depths of sampling (Supplementary Table S3), the relative contribution of phytoplankton to the total in situ absorption of light summed from 400 to 700 nm increased with depth to reach a maximum of 13% at 7.8 m, while CDOM absorbed 39% of the light energy at that depth, surpassing the contribution of water (35%). In contrast, phytoplankton only contributed 1.2% of total absorption at the surface of the shallow littoral zone whereas water itself contributed 71.6%.

According to the PARAFAC model, components C1, C2 and C3, which contributed around 30% of the lakewater CDOM, are terrestrially derived humic-like substances (Supplementary Fig. S5 and Table S4). C3 was found in lower concentrations at the surface of the water column and in the littoral zone, indicating low terrestrial inputs at the surface of the lake (Supplementary Fig. S6), as also suggested by the *S*_*289*_ index. The water tracks had a slightly higher proportion of terrestrial component C2 than in the water column (Supplementary Fig. S6). Components C4 and C5 correspond to protein-derived substances related to the amino acids tryptophan and tyrosine, typically associated with autochthonous primary production or other microbial processes. These two components contributed 60 to 79% of the total CDOM fluorescence in all samples, with the highest values in the light-exposed littoral zone (Supplementary Fig. S6). The water tracks contained 40% terrestrial components (sum of C1, C2 and C3), consistent with its higher CDOM content (*a*_*320*_; Supplementary Figs. S6 and S7).

**Figure 6:**
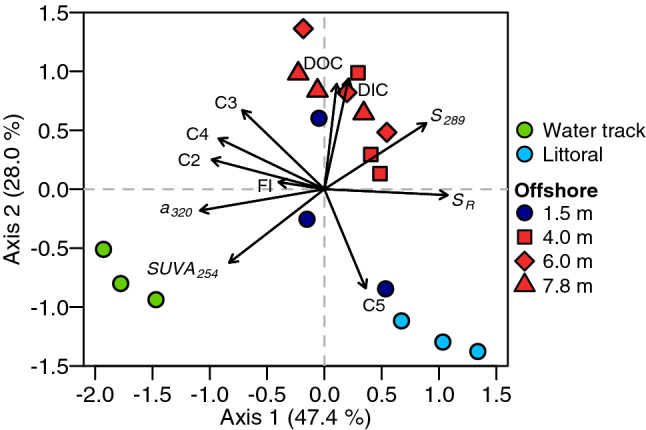
Principal component analysis of samples from Ward Hunt Lake, inflowing water tracks and the littoral zone in July 2017. DIC: dissolved inorganic carbon; DOC: dissolved organic carbon; *a*_*320*_: absorption coefficient at 320 nm; *SUVA*_*254*_: specific ultraviolet absorbance at 254 nm; *S*_*289*_: absorption slope parameter between 279 and 299 nm; *S*_*R*_: absorption slope ratio; *FI*: fluorescence index; C1 to C5: contribution of the five components identified by the PARAFAC model. Water tracks are subsurface flow paths draining the west side of the watershed.

A PCA analysis with all the optical data and carbon data (DOC and DIC concentrations) was performed to understand their contribution through the lake. The PCA showed a distinct separation of the littoral zone, mid-lake water column of Ward Hunt Lake and preferential subsurface flowpaths that are referred to in the permafrost literature as water tracks^12^ (Fig. 6). CDOM absorption (*a*_*320*_) and the SUVA index (DOC-normalized absorbance at 254 nm) were much higher in water tracks than in the water column (Supplementary Fig. S6). The largest *S*_*289*_ values in the water column were recorded towards the bottom of the lake while higher Chl *a* concentrations and *a*_*ph*_ values were obtained at 7.8 m (Fig. 5d; Supplementary Fig. S2e). Chl *a* concentrations and algal particle absorbance (*a*_*ph*_) were constant between 1.5 and 6.0 m (Fig. 5; Supplementary Fig. S2e) and non-algal particle absorption (*a*_*NAP*_) exceeded that by algal particles, with higher values at 1.5 and 7.8 m (Fig. 5a,d).

The lowest values of the slope ratio (*S*_*R*_) were observed in the water tracks, indicating higher DOM molecular weight, and the highest values were recorded in the littoral zone (Fig. 6). The water tracks had lower DOC and DIC concentrations than in the offshore water column, but higher absorption coefficients (Fig. 6; Supplementary Fig. S6). The higher specific absorption coefficients (absorption per unit DOC) co-occurred with lower *S*_*289*_ and *S*_*R*_ values, indicating a higher proportion of carbon from terrestrial sources in the water tracks.

## Discussion

**Figure 7:**
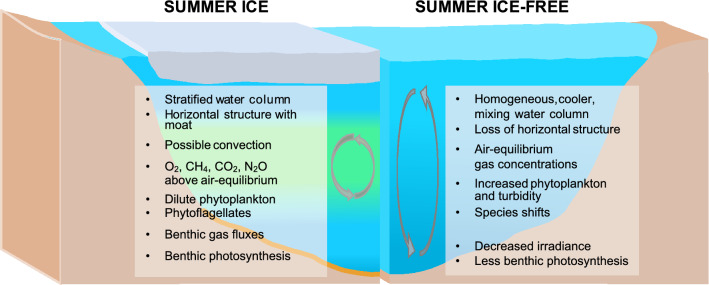
Summary of limnological observations in Ward Hunt Lake. Conditions under the mid-summer ice of Ward Hunt Lake and potential changes associated with loss of that ice in the future.

Our aim in the present study was to define the water column properties of a High Arctic freshwater lake capped by thick ice in summer. Given the accelerated warming taking place along this far northern coastline^13^, we also aimed to place these observations in the context of climate change, and to identify features that might be disrupted by summer ice loss. Figure 7 summarizes many of these observations from our field results reported here combined with information from previous reports on this lake, and considers potential changes that could occur after the loss of mid-summer ice in the future. Ward Hunt Lake was capped by > 4 m of summer ice in the past, but is now subject to ice-out at the end of summer in the warmest years, notably in 2016. With ongoing climate change in the High Arctic and the increased frequency of extreme warming events^13^, this loss of mid-summer ice may not be far into the future.

In brief, our observations show that despite its freshwater characteristics, the water column of Ward Hunt Lake was highly structured, with pronounced depth variations in limnological properties such as dissolved gases across the gradients of water density. The latter were maintained by solutes, which although in the freshwater range, varied sufficiently with depth to have effects on density that greatly exceeded those caused by temperature. This stabilizing effect of small changes in freshwater salinities has been described in Arctic ice-covered lakes in Alaska^14,15^, and would be lost by the loss of ice-cover and full water column circulation. This complete mixing would be favored by exposure to strong winds in the area, without the protection provided by ice-cover, and water temperatures below 4 °C that are conducive to cold monomixis. Other potential changes would include a cooler water column, ventilation of gases to the atmosphere, oxygenation of the sediments, loss of the moat and associated horizontal structure (described in Bégin et al.^16^), changes in phytoplankton composition, increased planktonic versus benthic production and increased turbidity (Fig. 7).

### Water column properties

Inverse thermal stratification occurred beneath the mid-summer ice of Ward Hunt Lake in all years of observation (Supplementary Figs S2a, S3a), with a stable boundary layer immediately beneath the ice. When the ice-cover completely disappeared in August 2016, wind induced mixing of the entire water column at that time led to pronounced heat loss^17^, and this ventilation of stored heat to the atmosphere is likely to occur after full ice loss in mid-summer in the future (Fig. 7), with implications for all temperature-dependent biogeochemical processes.

Oxygen profiles were similar in 2016 and 2017, with maximal concentrations above 150% air-equilibrium between 4 and 8 m. The homogeneous mid-water column concentrations of O_2_ as well as other gases are consistent with a convective mixing cell, which is observed in north temperate lakes in spring^8^ and here in Ward Hunt Lake extending into mid-summer. This penetrative convection can be halted by the density effect of small gradients in solute concentration (including possibly at the time of measurement), and horizontal convection along with currents induced by internal waves can also produce complex patterns in water column structure beneath the ice^15^. The presence of a moat (Supplementary Fig. S1) and the associated horizontal structure^16^ may contribute to the water column features observed here, but these effects would be lost with mid-summer ice loss (Fig. 7).

The mid-water column layer of oxygen supersaturation attained its highest value (180%) in mid-summer 2017, likely as a result of improved light availability for photosynthesis under the 1-year ice relative to the thicker and less transparent multi-year ice in the summers of 2015 and 2016. Oxygen supersaturation is a common feature of ice-capped polar lakes, for example ca. 300% of air-equilibrium in the photosynthetic maximum of Lake Fryxell, Antarctica^18^. Complete water column mixing in 2016 resulted in ventilation of oxygen to the atmosphere, and a decrease to air-equilibrium values^17^. This equilibration with the atmosphere is likely to occur for all gases under summer ice-free-conditions (Fig. 7).

### Phototrophic communities

Despite the presence of an ice-cover almost 2 m thick, up to 10% of PAR (400–700 nm) reached the bottom of the water column of Ward Hunt Lake and provided adequate energy for the growth of primary producer communities, particularly given the continuous daylight regime in summer. The fatty acid composition of seston in the lower water column suggested a high abundance of chrysophytes, which have been previously identified in the Ward Hunt Lake water column and in other Arctic lakes^19^. Their motility as well as likely mixotrophic capabilities may offer a competitive advantage over obligate phototrophs such as diatoms^20^ in the low light, low nutrient environment of Ward Hunt Lake. Abundant large colonies of the chrysophyte genus *Uroglena* were observed during summers 2016 and 2017, and continuous Chl *a* fluorescence records suggest their periodic migration in the water column according to in situ light fluctuations^17^. The presence of diatoms and dinoflagellates higher in the water column was suggested by the presence of the fatty acids C16:1n-7, C22:6n-3, and C20:5n-3, and could be a high quality food for consumers. These groups have been previously identified by microscopy in the littoral zone of this lake^19,21^. Diatoms have been conspicuously sparse or absent from previous analyses of Ward Hunt Lake phytoplankton samples from beneath the ice, although they occur in the open waters of the moat^16^. These fast sinking taxa may be more likely to thrive under full water column mixing with the loss of summer ice, while such conditions may cause the breakup of delicate chrysophytes such as the large *Uroglena* colonies, resulting in a shift of species (Fig. 7).

Benthic microbial mats are a common feature in Antarctic and Arctic lakes, where they can dominate overall ecosystem biomass and productivity^22,23^. The microbial mats sampled here all contained pigment stocks that were much larger than those in the overlying water column. The optical analysis showed that there were large changes in light quality as well as quantity with depth, and this spectral change with depth will favor spectral matching by the phytoplankton and microbial mats in their photosynthetic accessory pigments^24^.

### Greenhouse gas concentrations

Concentrations of CO_2_ in Ward Hunt Lake were well above air equilibrium values throughout the water column (Fig. 3). Carbon dioxide supersaturation is common in boreal lakes as respiration rates are generally higher than photosynthetic carbon fixation rates^25,26^, and this effect is also observed in many high latitude lakes and ponds^27,28^. This supersaturation is generally attributed to the mineralisation of carbon subsidies from allochthonous sources, but these inputs are likely to be small in Ward Hunt Lake. Here the supersaturation most probably derives from net heterotrophy over winter, with decomposition processes in the plankton and especially in the high biomass microbial mats. Bicarbonate ions dominate the relatively high DIC of the lake water^29^ and may be taken up by cyanobacterial mat photosynthesis in summer, to be partially released by bacterial decomposition in winter. The summer consumption of CO_2_ in the water column (271 mmol m^−2^) was only 24% that of oxygen production and a minimal fraction (2%) of the water column DIC stock (15 800 mmol C m^−2^ ), which could be related to the photosynthetic use of bicarbonate.

Concentrations of CH_4_ rose sharply with depth to extreme values that were three orders of magnitude above air-equilibrium at the bottom of the water column. Methane accumulation is common in ice-covered lakes^26^, and these values are within the range of concentrations reported in Arctic trough ponds^30^ and subarctic thermokarst ponds^28^. They are consistent with the anoxic conditions measured previously during winter in the bottom waters of this lake^16^.

Nitrous oxide concentrations were also above air-equilibrium, although not at the extreme levels found in some ice-capped Antarctic lakes^31^. This accumulation of N_2_O implies active nitrification under low oxygen conditions. Both CH_4_ and N_2_O concentrations dropped substantially over summer, consistent with active photosynthesis at the bottom of the water column at this time of year, and provision of oxygen for methanotrophy above the mats, along with more complete nitrification to nitrate and less production of N_2_O, which may be ultimately consumed by denitrifiers deep within the mats. In the absence of summer ice, these gradients would be lost, and greenhouse gases that accumulated during winter would be vented to the atmosphere at ice break-up and mixing in spring, without this opportunity for biogeochemical conversion^28^. The more oxygenated benthic environment may be less conducive to methane production, and colder water conditions may dampen all gas-producing processes.

### CDOM and under-ice spectral irradiance

The analysis of CDOM in Ward Hunt Lake indicated that it was derived mainly from autochthonous microbial sources, as we initially surmised. This oligotrophic waterbody lies in a sparsely vegetated polar desert catchment, and the reduced terrestrial influence is reflected in its low DOC and CDOM concentrations, as well as by the weak coloration of CDOM in the lake versus water tracks. The lake water column values of *a*_*320*_ of around 0.5–0.6 m^−1^ (Supplementary Fig. S6) are one to two orders of magnitude below those in subarctic tundra lakes and ponds that receive inputs from degrading permafrost soils in a well-vegetated region (lake water *a*_*320*_ values of 10–56 m^-1^)^32^. Similarly, the water column values of DOC-specific absorption (a*_CDOM_) of around 0.5 m^2^ g^-1^ are well below values found in Subarctic and Arctic rivers (2.5–4.2 m^2^ g^−1^)^33^, and are more similar to oceanic values (0.6 m^2^ g^−1^ in the offshore Arctic Ocean)^33^.

The low contribution by terrestrial sources to DOM in Ward Hunt Lake was further indicated by the PARAFAC analysis, which showed that protein-associated compounds contributed up to 79% of total DOM fluorescence. This is in striking contrast to thaw ponds in eroding peatland soils, where these components contributed only 27% of the DOM fluorescence^34^. Ward Hunt Lake values are more comparable to the range found in the open ocean, for example the Atlantic Ocean where these low molecular weight compounds can account for 93% of DOM fluorescence^35^. The sparse vegetation and its low productivity in the Ward Hunt Lake watershed is likely to limit the input of nutrients and plant degradation products to the lake, as in polar deserts elsewhere, making this lake an interesting end-member among lakes of the world for ongoing limnological analysis and monitoring.

Reflection and attenuation by the ice cover of Ward Hunt Lake blocked 40 to 60% of the incoming irradiance, with attenuation of longer wavelengths by the H_2_O molecules shifting the spectrum towards blue-green wavelengths (Fig. 4). Changes in the surface reflectivity (albedo) are likely to play a major role in controlling the under-ice irradiance. Ward Hunt Lake is rarely covered by snow in mid-summer, and by July each year the surface ice has begun to candle, which can increase light transmission^36^. Deeper in the water column, and despite its low concentration and weak coloration, DOM also played an important role in the underwater light regime of Ward Hunt Lake. Contrary to our hypothesis, although water was the primary light absorbing component in the ice and water column, CDOM was optically more important than phytoplankton. It was present in sufficient quantities to alter underwater spectral irradiance, producing a yellow-green light regime centred at 550 nm reaching the microbial mats. This might be partly associated with a small but highly colored terrestrial fraction, possibly derived from the water tracks, which had higher values of a*_320_. Water tracks are subsurface features that pass through and beneath mixed assemblages of terrestrial cyanobacteria, heterotrophic bacteria, lichens, mosses and some higher plants such as *Phippsia algida, Saxifraga oppositifolia* and *Carex* spp.^12^, likely picking up a mixture of microbial and plant-derived organic materials. Phytoplankton and non-algal particles played a relatively minor role in the absorption of photons in the lake water column, but may have contributed to attenuation via scattering. Contrary to expectation, CDOM was the main contributor to light absorption at lower wavelengths in the ultra-oligotrophic waters of Ward Hunt Lake, especially just below the ice (1.5 m) and near the lake bottom (7.8 m).

Large changes may occur in the underwater irradiance regime with ongoing climate warming and the loss of summer ice (Fig. 7). Although the ice cover reduces light at the top of the water column, this effect may be completely countered by an increase in water column attenuation, via several mechanisms. Increased mixing and increased nutrient inputs from a warmer, more biogeochemically and hydrologically active catchment may stimulate phytoplankton and this would increase light attenuation by phytoplankton pigments. Increased wind exposure and mixing may suspend sediments and cause shoreline erosion, leading to an increase in non-algal particulates. Evidence for this effect was seen in the open water period of 2016, when PAR attenuation values increased to^17^ 0.8 m^−1^. This would result in PAR at the bottom of the lake dropping from > 5% as measured here to 0.03% of incident PAR, which may preclude the development of microbial mats at these depths, and shift the balance of primary production more towards the phytoplankton community (Fig. 7). An additional effect moving the ecosystem in the same direction may be CDOM, which is an increasingly important component of Arctic freshwater ecosystems as more terrigenous inputs are expected in the future with accentuated precipitation, permafrost degradation and increased vegetation^35^; Ward Hunt Lake and other polar desert waterbodies may be especially sensitive to these changes.

## Conclusions

High latitude lakes are ice-bound ecosystems and are therefore vulnerable to ongoing contraction of the cryosphere. The physicochemical structure of Ward Hunt Lake in summer is fundamentally influenced by its ice-cover. A density stratified water column and accumulation of gases to well above air-equilibrium are made possible by the ice-impeded exchanges with the atmosphere. Moreover, the ice-cover limits the quantity of incoming light by a factor of two and attenuates longer wavelengths to a greater extent than UVR. With low DOC inputs from the watershed, carbon cycling in Ward Hunt Lake is essentially based on internal, autochthonous production, with microbial mats growing under a dim CDOM-influenced spectral irradiance regime. These biomass-rich mats likely play the dominant role in the production and consumption of greenhouse gases. In this and other polar lakes, vertical gradients in the under-ice water column reflect not only current conditions, but also the biogeochemical consequences of prolonged darkness, heterotrophy and anaerobic metabolism over the preceding winter.

With ongoing rapid warming at high northern latitudes^13^, mid-summer ice loss is likely to occur in the future. The resultant complete mixing of the water column will reconfigure Ward Hunt Lake, and other freshwater lakes of the extreme High Arctic. This will result in shifts in the magnitude of energy and gas exchanges with the atmosphere, and the accompanying variations in vertical structure will provide a sensitive guide to ongoing change.

## Materials and methods

### Study site

Ward Hunt Lake (83°05.226′N; 74°08.721′W; WGS84 map datum) is located 6 km off the northern coast of Ellesmere Island, within Quttinirpaaq National Park, Nunavut (Fig. 1). The lake has a maximum depth of 9.7 m and an area of 0.37 km^2^. The region experiences a polar desert climate characterized by a -16.6 °C mean annual temperature^37^, and 154.6 mm year^−1^ mean annual precipitation was recorded at Alert, 170 km to the east (1950–2017; Environment Canada, data available at http://climate.weather.gc.ca). Complete loss of the ice-cover was observed in 2011, 2012 and 2016^3,17^.

### Vertical profiling

Vertical profiling was performed in the deepest part of the lake (offshore zone) and at its margin (littoral zone) in July 2016 and 2017. Samples in the pelagic zone were collected at four depths: 1.5, 4.0, 6.0 and 7.8 m. Temperature and conductivity were recorded with a RBR Concerto profiler (RBR, Ottawa, Canada). Dissolved O_2_ and chlorophyll *a* (Chl *a*) profiles were recorded with a YSI-600QS probe in 2016 and a YSI-EXO2 in 2017 (YSI, Yellow Springs, OH). Salinity- and temperature-based density profiles were computed with the LIM toolbox for MATLAB^38^ integrating the major ion composition (including bicarbonate) of inflows measured in the watershed in 2014. Water for anions was filtered through 0.2 µm cellulose acetate filters (Advantec MFS, Dublin, CA) and both anions and cations were measured by ion chromatography (ICS-2000 Dionex Corporation, Sunnyvale, CA). The Brunt-Väisälä frequency (*N*) was calculated as: $N = \sqrt {g\left( {\frac{\Delta \rho }{{\Delta z}}} \right)/\overline{\rho }}$ , where *g* is the gravitational acceleration (9.8 m s^-2^), $\Delta \rho$ the difference in density between two layers of water, $\Delta z$ the distance between the two layers and $\overline{\rho }$ the maximum density of pure water (1000 kg m^−3^).

Downwelling (*E*_*d*_(*λ*)) and upwelling irradiance (*E*_*u*_(*λ*)) in the water column were measured with a Ramses ACC UV/VIS cosine-corrected probe (TriOS, Germany). Transmittance was calculated as the proportion of downwelling irradiance at a given depth relative to incident downwelling irradiance in air at the surface. Reflectance was expressed as the ratio of upwelling to downwelling irradiance at the same depth (*E*_*u*_(*λ*)/*E*_*d*_(*λ*)). Total energy was the sum of downwelling irradiance values (in mW m^-2^) for all measured wavelengths (278–720 nm). Diffuse attenuation coefficients (*K*_*d*_) were calculated between adjacent water layers with the equation: *K*_*d*_ = − ln (*E*_*2*_/*E*_*1*_) / (*z*_*2*_ – *z*_*1*_) where *E*_*1*_ is the irradiance measured above (depth *z*_*1*_ in m) and *E*_*2*_ is the irradiance measured below (*z*_*2*_).

For the greenhouse gas analyses, lake water was collected every meter between 2 and 10 m on 7 June and 16 July 2017, and immediately transferred to 2 L gas exchange water bottles. CO_2_, CH_4_ and N_2_O dissolved in the water were equilibrated with 20 mL of ambient air by shaking vigorously for 3 min, and the headspace gas then transferred to Exetainer vials (Labco, United Kingdom) with butyl rubber septa; our previous tests showed that a vacuum was maintained (no leakage) in these vials for at least one year and that they were therefore suitable for long term storage of gas samples. The Ward Hunt Lake samples were analyzed 3 months after collection by gas chromatography with a Trace 1310 GC (Thermo Fisher Scientific, U.S.A.) that was calibrated with gas standards from Merck Millipore (Analytical Grade; Sigma-Aldrich, Canada) for the ranges 0–5000 ppm (CH_4_, low range),0–45,000 ppm (CH_4_, high range), 10–10,000 ppm (CO_2_) and 0–1 ppm (N_2_O). The dissolved gas concentrations were calculated as described in Prėskienis et al.^39^, taking into account the headspace gases and water volume ratio. Major ions concentrations were dilute in Ward Hunt Lake (maximum of 0.3 g L^-1^), and therefore no correction was made for salinity since it was too low to have a measurable effect on gas solubility^40^.

### CDOM, pigments, and fatty acids

Lake water for colored dissolved organic matter (CDOM), dissolved organic carbon (DOC) and dissolved inorganic carbon (DIC), in vivo absorbance and pigment analysis was collected in July 2017 at 4 depths (1.5, 4.0, 6.0 and 7.8 m from the top of the ice-cover) with a Limnos sampler (Limnos, Turku, Finland). Samples from preferential subsurface flow paths known as water tracks are abundant on the western shore of the lake. The water from these tracks was directly collected where it was seeping up to the surface near the lake shore. Water for CDOM, DOC and DIC was filtered through 0.2 µm cellulose acetate filters (Advantec MFS, Dublin, CA) pre-rinsed with Milli-Q water and stored in the dark in glass bottles at 4 °C until analysis. DOC and DIC concentrations were measured by infrared detection in a carbon analyzer (TOC-VCPH, Shimadzu, Kyoto, Japan) after catalytic combustion. Absorbance (*A*_*λ*_) of CDOM was measured through 10 cm quartz cuvettes from 200 to 800 nm at 1 nm interval using a Varian Cary 100 dual-beam spectrophotometer (Varian Inc., Santa Clara, CA). Following the protocol described by Helms et al.^41^, we conducted a null point correction by the subtracting the mean *A*_*λ*_ from 750 to 800 nm to the complete spectra after the subtraction of the blank spectrum. Absorption coefficients were calculated as *a*_*λ*_ = 2.303 * *A*_*λ*_ / *L*, where *a*_*λ*_ is the absorption coefficient (m^-1^) at the wavelength *λ*, *A*_*λ*_ is the absorption at the wavelength *λ*, and L is the length of the cuvette (m). The specific ultraviolet absorbance at 254 nm (*SUVA*_*254*_) was used as an indicator of CDOM aromaticity^42^. The indexes *S*_*289*_, corresponding to the slope parameter between 279 and 289 nm, and *S*_*R*_, the ratio between the slope parameters *S*_*285*_ (275–295 nm) and *S*_*375*_ (350–400 nm), were calculated as in Loiselle et al.^43^ and Helms et al.^41^.

The fluorescence intensity of dissolved organic matter (DOM) was measured with a Cary Eclipse spectrofluorometer (Agilent, Santa Clara, California) from 300 to 560 nm (2 nm increments) with excitation from 250 to 450 nm, at 10 nm increments. The fluorescence index (FI) was used as an indicator of the origin of fulvic acids and was expressed as the ratio of fluorescence emission intensities at 450 nm and 500 nm exposed to excitation at 370 nm^44^. The fluorescence excitation and emission matrices (EEMs) were divided in DOM components with a parallel factor model (PARAFAC) using MATLAB v R2013a (MathWorks, Natick, Massachusetts) as in Murphy et al.^45^. In addition to our 15 samples from Ward Hunt Lake, 100 samples from Wauthy et al.^34^ were included to run the model. The EEMs were corrected for Raman and Rayleigh scattering and for inner filter effects, and were standardized to Raman units with the FDOMcorr 1.4 Toolbox^46^. The maximum fluorescence ([Cx]) obtained by the model for each component was summed to quantify the total fluorescence (F_T_). The proportion of contribution of every component was then calculated for each sample as in Wauthy et al.^34^: %Cx = ([Cx]/F_T_) × 100.

A principal component analysis (PCA) was performed to understand the spatial distribution of CDOM with the standardized fluorescence and absorbance indicators using the *rda* function of the *vegan* package in *R*^47^. As the components C1 to C5 obtained via the PARAFAC analysis are expressed as percentages of contribution to the CDOM composition and are mathematically dependent, an additive log-ratio transformation for compositional data was performed with C1 as the denominator variable (*alr* function from the *compositions* package).

Lake water for seston absorbance measurements was filtered through 25 mm GF/F filters that were preserved at -80 °C until analysis. The optical density of the material collected on the filters (in vivo) was measured from 300 to 720 nm in a Varian Cary Bio 300 dual-beam spectrophotometer (Agilent, Santa Clara, California) equipped with an integrating sphere (Labsphere Inc., North Sutton, NH) and processed as described in Bégin et al.^16^. Absorption coefficients for pure water were obtained from the IOCCG Protocol Series^48^.

Lake water for pigment analysis was filtered through 25 mm GF/F filters that were preserved at -80 °C until analysis. Pigments were extracted from the filters with warm methanol 95% and measured with high pressure liquid chromatography (HPLC), as described in Bonilla et al.^21^. Pigments were associated with phototrophic groups according to Roy et al.^49^ and Bonilla et al.^21^. Microbial mats were collected in July 2015 in the offshore zone at around 9 m depth using a Mini-Glew corer^50^. The upper 3–4 mm of the core containing live cells (as indicated by light microscopy of fresh samples within 36 h of collection) were kept frozen at -80 °C until analysis. Pigments were analyzed by the same HPLC method described above, after a succession of four extractions on lyophilized samples with 90% acetone/10% water.

Seston samples for fatty acid analyses were collected at 1.5 and 7.8 m under the ice-cover with the Limnos sampler and preserved on GF/F filters. Zooplankton samples were collected with a tow net (63 µm mesh) and transferred onto GF/F filters. Seston and zooplankton samples were kept frozen and freeze-dried. Fatty acids were extracted following a one-step transmethylation in methanol:toluene:acetyl chloride (4000:1000:125) at 90 °C for 20 min; the fatty acid methyl esters (FAMEs) were then extracted with water and hexane, and quantified by gas chromatography-mass spectrometry (GC–MS) as described in Schneider et al.^51^. Analyses focused on the unsaturated FA C16:1n-7, C18:2n-6, C18:3n-3, C20:4n-6, C20:5n-3, C22:6n-3 and C24:1n-9 as biomarkers of different phytoplankton groups, the saturated FA C20:0, C22:0, and C24:0 as terrestrial biomarkers and the branched-chained saturated FA aC15:0, iC15:0, iC16:0 and iC17:0 as bacterial biomarkers^9,10,52^.

## Supplementary Information

Supplementary Information 1.

## Data Availability

Data are archived in the online, open access data repository Nordicana D (http://www.cen.ulaval.ca/nordicanad/en_index.aspx).
