# Supplementary material for: Water column gradients beneath the summer ice of a High Arctic freshwater lake as indicators of sensitivity to climate change
Source: Sci Rep. 2021 Feb 3;11:2868. doi: 10.1038/s41598-021-82234-z (PMC7858640; doi:10.1038/s41598-021-82234-z)
Supplement: Supplementary file 1 — Supplementary Information 1. [file 41598_2021_82234_MOESM1_ESM.docx]

## Supplementary material

# Water column gradients beneath the summer ice of a High Arctic freshwater lake as indicators of sensitivity to climate change

Paschale N. Bégin, Yukiko Tanabe, Milla Rautio, Maxime Wauthy, Isabelle Laurion, Masaki Uchida, Alexander I. Culley, and Warwick F. Vincent.

**Table S1.** Major ions in **surface** and bottom waters at the central sub-ice site (S4) of Ward Hunt Lake. Values are means of triplicates, with SE in parentheses. SpC: specific conductivity.

|  | SpC |  |  | Ion concentrations (mg L^-1^) | | | | | | | |
| --- | --- | --- | --- | --- | --- | --- | --- | --- | --- | --- | --- |
|  | (µS cm^-1^) | pH | T (°C) | HCO_3_^-^ | SO_4_^2-^ | NO_3_^-^ | Ca^2+^ | K^+^ | Mg^2+^ | Na^+^ |  |
| Surface | 182 | 7.41 (0.14) | 0.15 | 47.7 (9.1) | 5.6 (0.8) | 0.5 (0.23) | 33.8 (0.5) | 0.5 (0.05) | 5.4 (0.2) | 5.4 (2.0) |  |
| Bottom | 276 | 7.39 (0.11) | 4.9 | 85.0 (1.5) | 6.0 (0.3) | 0.4 (0.03) | 44.3 (2.4) | 0.6 (0.03) | 6.6 (0.4) | 4.3 (0.2) |  |

Methods: Water for anions was filtered through 0.2 µm cellulose acetate filters (Advantec MFS, Dublin, CA) and both anions and cations were measured by ion chromatography (ICS-2000 Dionex Corporation, Sunnyvale, CA).

**Table S2. Gas storage in the water column of Ward Hunt Lake.** Carbon dioxide (CO_2_), methane (CH_4_), nitrous oxide (N_2_O) and oxygen (O_2_) concentrations were integrated over the depth range from 2 to 9 m (% change in storage between dates in parentheses).

| Gas | June 7 | July 16 | Difference |
| --- | --- | --- | --- |
| CO_2_ (mmol m^-2^) | 554.1 | 282.9 | -271 (-49%) |
| CH_4_ (mmol m^-2^) | 3.71 | 1.73 | -1.98 (-53%) |
| N_2_O (µmol m^-2^) | 91.5 | 73.9 | -17.6 (-19%) |
| O_2_ (mol m^-2^) | 3.58 | 4.72* | +1.15 (+32%) |

* Profiling on 12 July 2017

**Table S3. Relative contribution of optically active constituents to in situ absorption in Ward Hunt Lake**. The spectral absorption values were multiplied by the downwelling irradiance at each wavelength at the depth of sampling and summed to calculate % contribution to total in situ absorption over the PAR waveband from 400 to 700 nm.

| Constituents |  | Offshore zone | | | |  | Littoral zone |
| --- | --- | --- | --- | --- | --- | --- | --- |
|  |  | 1.5 m | 4.0 m | 6.0 m | 7.8 m |  | Surface |
| *a_w_* |  | 43.8 | 49.7 | 43.9 | 34.7 |  | 71.6 |
| *a_CDOM_* |  | 37.9 | 32.0 | 37.9 | 39.0 |  | 17.7 |
| *a_NAP_* |  | 15.3 | 11.5 | 10.5 | 13.3 |  | 9.5 |
| *a_ph_* |  | 3.1 | 6.8 | 7.7 | 13.0 |  | 1.2 |

**Table S4. Components of the PARAFAC fluorescence analysis.** Spectral maxima of the five components identified by the PARAFAC model, number of matches with the *OpenFluor* database (minimum similarity 0.95), assignment of sources and origin. Secondary peaks of excitation are presented in brackets.

| **Component** | **Maximum excitation** | **Maximum emission** | **Number of matches^a^** | **Assigned of sources** | **Origin^b^** |
| --- | --- | --- | --- | --- | --- |
| C1 | <260(350) | 490 | 24 | Humic-like | Terrestrial^1,2,3^ |
| C2 | <260(310) | 418 | 26 | Humic-like | Terrestrial^4,5,6,7^ |
| C3 | <260 | 436 | 6 | Humic-like, fulvic acid | Sedimentary^8,9,10^ |
| C4 | 290 | 350 | 10 | Protein, tryptophan-like | Autochthonous^1,3,8^ |
| C5 | 270 | 304 | 7 | Protein, tyrosine-like | Autochthonous^3,4^ |

^a^ Test performed with *OpenFluor* on March 2, 2018 (http://www.openfluor.org).
^b^ See references at the end of the document

**Figure S1. Ice-free water zone (moat) on the northern and western shores of Ward Hunt Lake in mid-summer 2015, 2016 and 2017.**

**Figure S2. Physicochemical profiles in Ward Hunt Lake.** a) temperature (Temp.), b) specific conductivity (Cond.), c) density, d) dissolved oxygen (O_2_ as % air-equilibrium), and e) chlorophyll *a* (Chl *a*) profiles in the water column of Ward Hunt Lake on 14 July 2016 (black) and 12 July 2017 (red). The Chl *a* profile in 2016 (squares and full line) was measured at four depths with high pressure liquid chromatography, and in 2017 (dashed line) by *in vivo* Chl *a* fluorescence with an EXO2 profiler (YSI, Yellow Springs, OH). The horizontal lines represent the depth of the ice cover.

**Figure S3. Physicochemical profiles in Ward Hunt Lake from 2010 to 2017**. a) Water temperature, b) dissolved oxygen expressed in % air equilibrium, and c) specific conductivity.

**Figure S4. Principal coordinate analysis of the fatty acid assemblages in Ward Hunt Lake.** Zooplankton were collected by net haul over the whole depth of the mid-lake water column. The arrows represent the contribution of the five most influential fatty acids on the distribution of sites. *The length of the C16:1n-7 arrow was divided by 10 compared to the other arrows.

**Figure S5.** **CDOM fluorescence components.** Fluorescence excitation and emission signatures of the five components identified by the PARAFAC model.

**Figure S6. CDOM and associated chemical properties of Ward Hunt Lake and inflows.** Measurements were made on samples from inflowing water tracks (WT) and the littoral zone of the lake (Litt.) and the mid-lake water column (4 depths). DOC and DIC: dissolved organic and inorganic carbon; *FI*: fluorescence index; *a_320_*: absorption coefficient at 320 nm; *a*_320_*: absorption coefficient at 320nm normalized by DOC concentration*; SUVA_254_* : fluorescence index at 254 nm; *S_289_*: absorption slope parameter between 279 and 299 nm; *S_R_*: absorption slope ratio. Values are means for triplicates with SE; different letters represent significant differences between the zones and depths according to a Tukey HSD multiple comparison following a significant ANOVA with the p-values adjusted by the Benjamini-Hochberg procedure ($\alpha$=0.05). Results of ANOVA comparisons of sites: DOC: F_5,12_ = 9.98 P = 0.001; DIC: F_5,12_ = 22.05, P < 0.001; *FI*: F_5.12_ = 0.398, P = 0.841; *a_320_*: F_5,12_ = 19.34, P <0.001; *a*_320_*: F_5,12_ = 19.02, P <0.001; *SUVA_254_*; F_5,12_ = 20.55, P < 0.001; *S_289_*: F_5,12_ = 58.91, P <0.001; *S_R_*: F_5,12_ = 36.79, P <0.001.

**Figure S7. CDOM fluorescence components in Ward Hunt Lake and inflows.** The % contribution of the five components identified by the PARAFAC model (C1 to C5) in the water tracks (WT), littoral zone (Litt.) and mid-lake water column (4 depths) of Ward Hunt Lake. Values are means for triplicates with SE; different letters represent significant differences between the zones and depths according to a Tukey HSD multiple comparison following a significant ANOVA with the p-values adjusted by the Benjamini-Hochberg procedure ($\alpha$=0.05). Results of ANOVA comparisons of sites: C1: F_5,12_ = 1.03, P = 0.442; C2: F_5,12_ = 3.28, P = 0.043; C3: F_5,12_ = 11.48, P < 0.001; C4: F_5,12_ = 1.761, P = 0.196; C5: F_5,12_ = 7.17, P = 0.003.

## References

1. Catalá, T. S. et al. Turnover time of fluorescent dissolved organic matter in the dark global ocean. Nat. Commun. 6, 5986; 10.1038/ncomms6986 (2015).

2. Shutova, Y., Baker, A., Bridgeman, J. & Henderson, R. K. Spectroscopic characterisation of dissolved organic matter changes in drinking water treatment: From PARAFAC analysis to online monitoring wavelengths. Water Res. 54, 159–169 (2014).

3. Murphy, K. R. et al. Organic matter fluorescence in municipal water recycling schemes: Toward a unified PARAFAC model. Environ. Sci. Technol. 45, 2909–2916 (2011).

4. Yamashita, Y., Panton, A., Mahaffey, C. & Jaffé, R. Assessing the spatial and temporal variability of dissolved organic matter in Liverpool Bay using excitation–emission matrix fluorescence and parallel factor analysis. Ocean Dyn. 61, 569–579 (2011).

5. Garcia, R. D., Reissig, M., Queimaliños, C. P., Garcia, P. E. & Dieguez, M. C. Climate-driven terrestrial inputs in ultraoligotrophic mountain streams of Andean Patagonia revealed through chromophoric and fluorescent dissolved organic matter. Sci. Total Environ. 521–522, 280–292 (2015).

6. Graeber, D., Gelbrecht, J., Pusch, M. T., Anlanger, C. & von Schiller, D. Agriculture has changed the amount and composition of dissolved organic matter in Central European headwater streams. Sci. Total Environ. 438, 435–446 (2012).

7. Yamashita, Y., Maie, N., Briceño, H. & Jaffé, R. Optical characterization of dissolved organic matter in tropical rivers of the Guayana Shield, Venezuela. J. Geophys. Res. Biogeosci. 115, G00F10; 10.1029/2009JG000987 (2010).

8. Osburn, C. L. & Stedmon, C. A. Linking the chemical and optical properties of dissolved organic matter in the Baltic–North Sea transition zone to differentiate three allochthonous inputs. Mar. Chem. 126, 281–294 (2011).

9. Kothawala, D. N., von Wachenfeldt, E., Koehler, B. & Tranvik, L. J. Selective loss and preservation of lake water dissolved organic matter fluorescence during long-term dark incubations. Sci. Total Environ. 433, 238–246 (2012).

10. Osburn, C. L., Wigdahl, C. R., Fritz, S. C. & Saros, J. E. Dissolved organic matter composition and photoreactivity in prairie lakes of the U.S. Great Plains. Limnol. Oceanogr. 56, 2371–2390 (2011).
